# Supplementary material for: Photothermal-assisted antibacterial application of graphene oxide-Ag nanocomposites against clinically isolated multi-drug resistant Escherichia coli
Source: R Soc Open Sci. 2020 Jul 22;7(7):192019. doi: 10.1098/rsos.192019 (PMC7428222; doi:10.1098/rsos.192019)
Supplement: SI_RSOS-192019_R1.docx [file rsos192019supp1.docx]

Photothermal-assisted antibacterial application of GO-Ag nanocomposites against clinical isolated MDR *Escherichia coli*

Yuqing Chen^1,†^, Wei Wu^2, †^, Zeqiao Xu^3^, Cheng Jiang^4^, Shuang Han^5^, Jun Ruan^3,*^, Yong Wang^3,*^

^1^ Children’s ENT Department, The Affiliated Wuxi Matemity and Child Health Care Hospital of Nanjing Medical University, 214122 Wuxi, People’s Republic of China

^2^Cardiothoracic Surgery Department, The Affiliated Wuxi No. 2 People’s Hospital of Nanjing Medical University, 214002 Wuxi, People’s Republic of China

^3^Urology Surgery Department, and ^4^Department of Laboratory Medicine, The Affiliated Wuxi People’s Hospital of Nanjing Medical University, 214122 Wuxi, People’s Republic of China

^5^School of Biotechnology, Jiangnan University, 214122 Wuxi, People’s Republic of China

^*^author for correspondence: Jun Ruan, ruanjun818@sina.com; Yong Wang, wy8486@sina.com

^†^Yuqing Chen and Wei Wu contributed equally to this work

**Keywords:** *in vitro*, MDR *E. coli*, graphene oxide-silver, photothermal treatment

*Author for correspondence ().

†Present address:


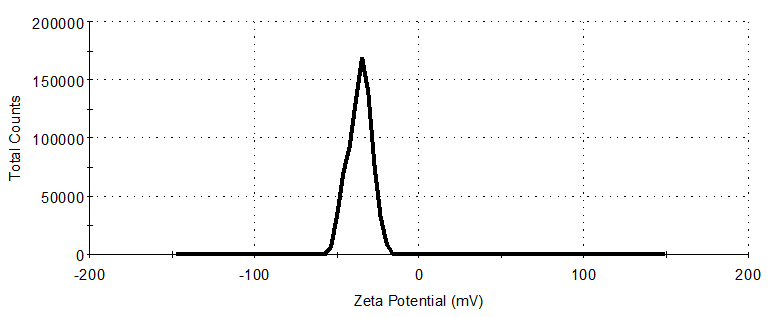


**Figure S1. Zeta potential distribution of GO dispersed in water.**

Zeta potential was measured using instrument named Zetasizer Nano (ZS90, Malvern). Zeta potential of GO was -36.1 ± 6.98 mV.

**
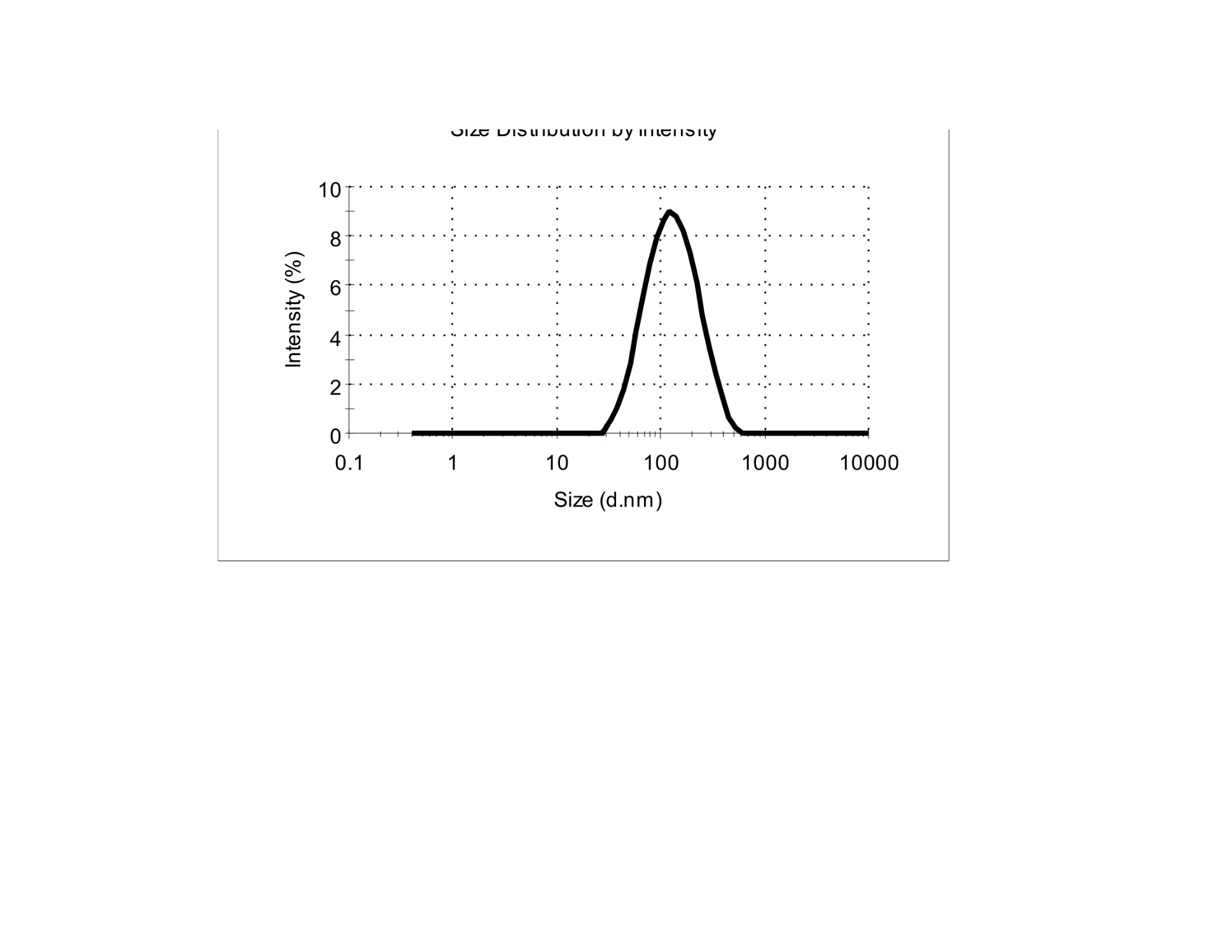
**

**Figure S2. Size distribution of GO-Ag nanocomposites dispersed in water.**

GO-Ag nanocomposites diameter were measured using Dynamic Light Scattering (DLS) method. Diameter of GO-Ag was 147.4 ± 85.16 nm.

**Figure S3. Heating curves of GO and GO-Ag at different power density.**

808 nm laser irradiation was conducted on GO and GO-Ag dispersed in LB broth at room temperature. Concentration of GO and GO-Ag were 3 µg mL-1 and 7 µg mL-1 respectively, and temperature was recorded by thermal camera.
