# Supplementary figures and images for: Photothermal-assisted antibacterial application of graphene oxide-Ag nanocomposites against clinically isolated multi-drug resistant Escherichia coli
Source: R Soc Open Sci. 2020 Jul 22;7(7):192019. doi: 10.1098/rsos.192019 (PMC7428222; doi:10.1098/rsos.192019)

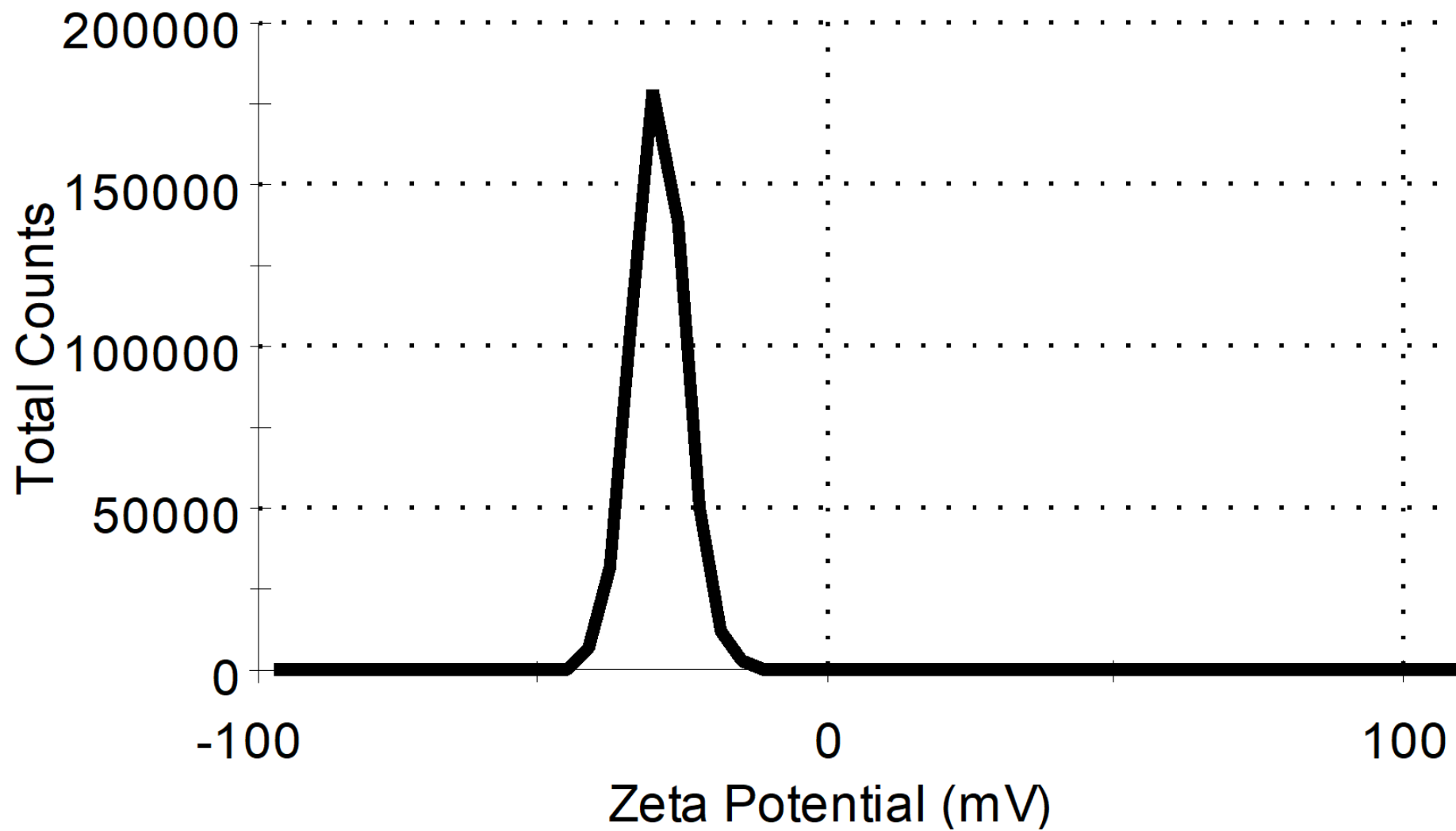

Supplement: Figure S1.pdf [file rsos192019supp2.pdf]

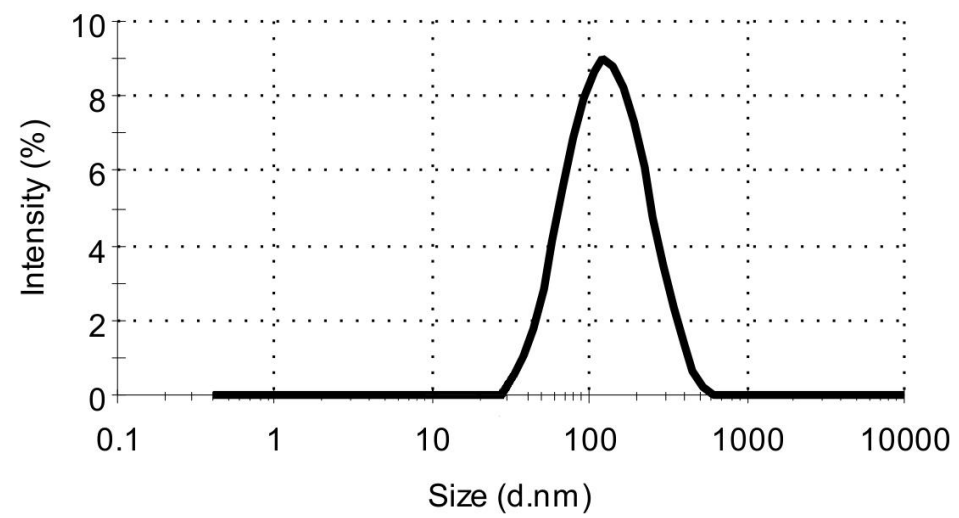

Supplement: Figure S2.pdf [file rsos192019supp3.pdf]

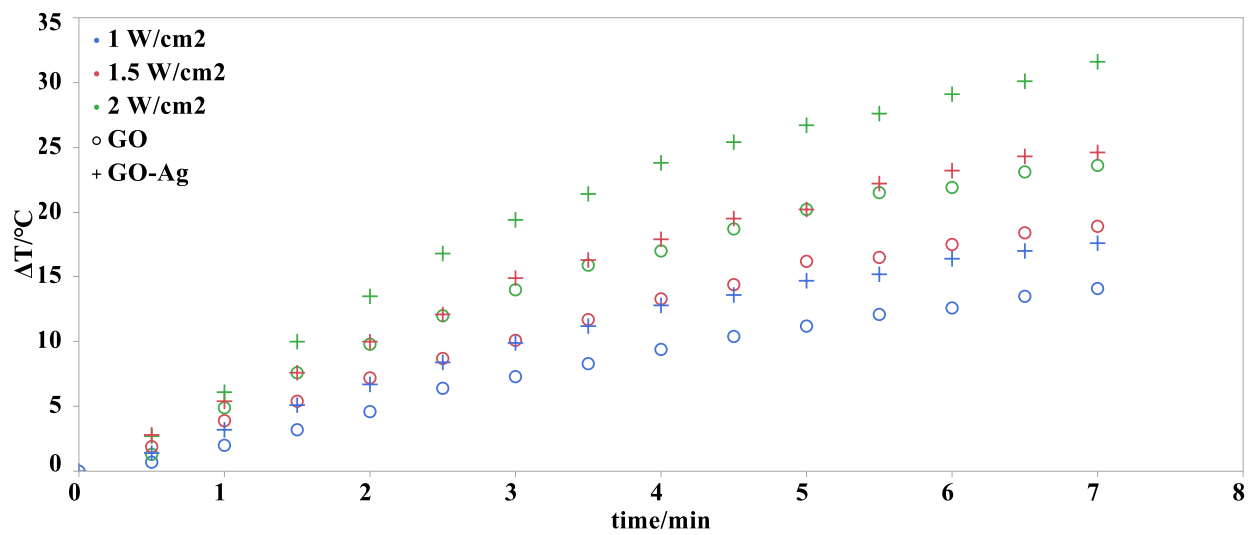

Supplement: Figure S3.pdf [file rsos192019supp4.pdf]
